# Conceptualization, operationalization, and content validity of the EQOL-questionnaire measuring quality of life and participation for persons with disabilities

**DOI:** 10.1186/s12955-018-1024-6

**Published:** 2018-10-11

**Authors:** Louise Norman Jespersen, Susan Ishøy Michelsen, Bjørn Evald Holstein, Tine Tjørnhøj-Thomsen, Pernille Due

**Affiliations:** National Institute of Public Health, University of Southern Denmark, Studiestræde 6, 1455 Copenhagen K, Denmark

**Keywords:** Questionnaire, Operationalization, Conceptualization, Disability, Quality of life, Participation, Content validity, Measurement, Instrument

## Abstract

**Background:**

Measurement of quality of life demands thoroughly developed and validated instruments. The development steps from theory to concepts and from empirical data to items are sparsely described in the literature of questionnaire development. Furthermore, there seems to be a need for an instrument measuring quality of life and participation in a population with diverse disabilities. The aim of this paper was to present and discuss the initial steps in the development of the Electronic Quality of Life questionnaire (EQOL).

**Methods:**

The development of EQOL included six steps: 1) Establishing conceptual understanding; 2) Development of interview guides which build on the conceptual understanding; 3) Qualitative interviews of 55 participants (10–40 years old) with different types and severities of disabilities; 4) Conceptualization of domains identified in the qualitative data through thematic analysis; 5) Operationalization of the identified domains into items and; 6) Evaluation of content validity of the first version of the EQOL-measure. Content validity was examined by cognitive interviews with participants in the target group as well as by continuous feedback from an advisory board.

**Results:**

We identified six domains (*function and health, environment (physical and social), social network, wellbeing, occupation, and managing strategies*) based on themes derived from the qualitative interviews and on conceptual discussions within the author group. These domains were incorporated in a conceptual model and items were generated to measure the content of each domain. Participants expressed satisfaction with EQOL but most participants felt that there were too many items.

**Conclusions:**

In total, 191 items were included in the questionnaire. Participants felt that the EQOL-questionnaire was relevant to their quality of life and participation. We have shown that it is possible to include quality of life and participation for people with various disabilities in one instrument. Although capturing less detail than a condition specific instrument, EQOL includes aspects perceived important for people with disabilities who are not included in general surveys. This is relevant when for example evaluating environmental adaptations and when comparing populations with various disabilities.

## Background

The concept of quality of life has over the past decades been a focus area within disability research as it provides important information about the life of the individual above biological and medical information [1–3].

Traditionally, clinical outcome measures have focused on physical symptoms, recurrence of events and mortality [4]. However, quality of life is the intuitive goal of all healthcare interventions [5], and the concept is becoming widely recognized as an important outcome in a variety of medical conditions, such as diabetes [4], cardiovascular disease [6], depression [7] and cancer [8]. Also, within disability research, it has been suggested that quality of life and participation should be considered the key outcomes [9, 10]. It is widely accepted to view quality of life as a multidimensional construct, which includes physical, mental and social dimensions [11].

A global disability prevalence of 14% has been estimated, based on difficulty in seeing, moving around, remembering/concentrating, and self-care [12]. Population groups with diverse disabilities are known to account for a great number of wide ranging health service needs [13] and adults with activity limitations report poorer wellbeing compared to those without activity limitation [14]. Research on adolescents with cerebral palsy able to self-report, show that they have a significantly lower quality of life in the domain of ‘social support and peers’, but a similar or better quality of life in nine other domains assessed [15]. We also know, that mental and physical conditions are strongly associated with social participation restrictions [16] and that adolescents with cerebral palsy participate less in physical and social activities than others [17, 18]. Limited social participation was also found among middle aged and older adults with multiple physical and mental disabilities [16]. However, little is known about the quality of life and participation in populations with diverse disabilities. This may be due to a lack of measures relevant to this wide-ranging and often undefined population.

Typically, questionnaires targeting disabilities are developed as either condition specific or generic [19]. Condition specific questionnaires can provide detailed information on issues important to individuals with specific conditions, diseases or symptoms [20]. In general, specific measures are more responsive in terms of measuring change in the quality of life of their target population compared to generic measures [21]. However, their specificity makes comparisons across disabilities difficult because questions relating to a particular condition may be difficult or irrelevant to answer for others without that disability.

Generic questionnaires address common dimensions across a wide range of populations [19], and do not refer to any specific illness or disability. While they allow for comparisons across populations with and without disabilities, they may not address issues important to particular patient groups, e.g. symptoms of disease and treatment of side effects. [22]. Also, generic quality of life instruments often include a measure of functioning or impairment [23]. If this measure is included as part of quality of life it may result in a systematically lower quality of life score among persons with physical disability or chronic illness [24, 25]. Examples of widely used generic quality of life measures are the WHOQOL-BREF [26] and the Quality of Life Scale [1]. Some measures, such as Quality of Life Index by Ferrans and Powers (1992) [27] are constructed as a generic core and a number of modules for specific illnesses or conditions.

Often a population of people with disabilities is not limited to one or a few types of disabilities. For example, in general population surveys, residential homes, municipalities, and hospitals, the population of interest is likely to vary in terms of type and severity of disability. To measure quality of life and participation in such populations, it is essential to assess these concepts in ways that are perceived relevant across a wide range of disabilities. This is to ensure valid results, as well as acceptability of the aspects addressed and further to allow for comparability across disabilities. Cross-disability assessments of quality of life and participation are useful in order to inform treatments, interventions, and environmental adaptations aiming to improve quality of life and reduce participation restrictions in populations with diverse disabilities.

Many generic instruments fail to include essential quality of life aspects important to individuals living with disabilities because they are based on what health professionals or the general populations consider important to quality of life [28, 29]. Therefore, perspectives from the target population obtained through qualitative interviews provide a good basis for developing a questionnaire [30]. When extracting information from qualitative interviews into themes it is crucial to be precise and explicit about which aspects of the obtained information that are included in each theme [31]. Furthermore, it is important that the items chosen to measure a certain theme reflect these inclusions [32]. Currently there is a wide range of measures aiming to measure quality of life. Descriptions of the development of these measures often include evaluation of the validity but lack details regarding the step from empirical data to items [31].

For instance in the WHOQOL disability module it is clear that focus groups were conducted in 15 centers around the world and that suggestions about which facets and items to add to the existing WHOQOL were translated into English [33]. They further describe that 20 additional items were pilot tested, but the methods used in this item selection process are not described in detail. A paper on the development of a participation questionnaire provides a section on item generation, but does not describe exactly how the items were chosen [34]. Another paper reports the development of a questionnaire for identification of children with chronic conditions. The authors describe the development as well as validity and reliability of the measure [35], and that items were generated based on existing measures, literature and interviews, and they present an example of the operationalization of domains, but also, this study lacks details about how the information obtained from the interviews were transformed into items.

Often the content validity of quality of life measures is assessed only by clinical experts [36]. Assessment of content validity by experts in the field may provide information on whether the questions measure the construct(s) you intend them to measure [37]. However, interviewing individuals from the target population about their perceptions of an questionnaire may provide important insight about the degree to which the instrument provides an adequate reflection of the construct [37] and this is an important addition to the expert assessment.

**Fig. 1:**
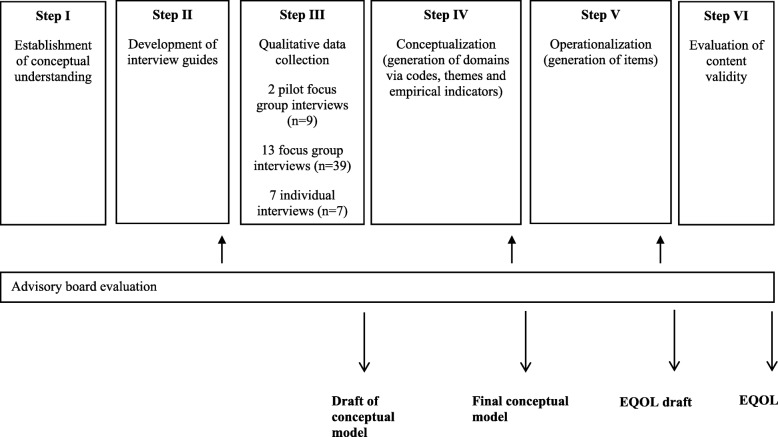
Flow chart on the development of EQOL

The aim of this paper was therefore to present and discuss the initial development of the Electronic Quality of Life (EQOL) questionnaire (Fig. 1) attempting to measure quality of life and participation across people with diverse disabilities. Conceptualization of information derived from the qualitative interviews and operationalization of the ensuing domains into items was a special focus of the study. Another objective was to explore the content validity of the EQOL-measure. Evaluation of EQOLs psychometric properties is beyond the scope of this paper.

The development of EQOL is presented in six steps (Fig. 1): 1) Establishing *conceptual understanding*; 2) Development of *interview guides* which reflect the conceptual understanding; 3) Interview guide-based *qualitative interviews* of participants with different types and severities of disabilities; 4) *Conceptualization* of domains by way of codes, themes and empirical indicators identified in the qualitative data; 5) *Operationalization* of the identified domains into items; and 6) evaluation of *content validity* of the first version of the EQOL-questionnaire.

### Definition of terms

The concepts of quality of life and participation provided the basis for this study. In order to describe the development of a questionnaire measuring these concepts, we used several specific terms during the development process. *Concepts and sub-concepts* referred to quality of life and participation inspired by the literature (Table 1). *Themes* designated the results of the thematic analysis on the qualitative interviews. *Domains* were compiled themes incorporated in the conceptual model. *Empirical indicators* were specific experiences chosen to represent a domain and *items* were the questions developed to measure the empirical indicators.

**Table 1:** Concepts, sub concepts, and questions included in the interview guide

Step I			Step II
Concept	Sub-concepts	Relevant references	Question
Quality of life	Perception of the quality of life construct High and poor quality of life	Psychological/spiritual domain [62] WHO definition [26]	Can you tell me how you perceive the term quality of life? Please collaborate on sorting these photos in two piles. One representing a good quality of life, the other representing a poor quality of life. Provide each pile with three headlines. If not already answered through the exercise: What makes your quality of life good? What makes your quality of life poor?
Participation	Perception of the participation construct Prioritizing participation (What do you prefer)	ICF [38] ICF, Activity and participation [38] Health and Functioning [62]	Can you tell me about how you perceive participation? What types of participation are important to you?
Perception of environment	People around you	ICF, Environmental factors [38] Family domain, health and functioning domain, social and Economic domain [62] Environment [63] Interpersonal relations, social inclusion, rights [46]	How do your family and friend affect your quality of life and participation? How does the contact to the health system affect your quality of life and participation?
	Inclusion or differentiation		Tell me about the differences in being with others who also has a disability and being with others who have not got a disability. If you experience discrimination related to your disability; how do you experience it?
	Physical surroundings		What impact has the physical environment on your quality of life and participation? Can you tell me about whether it is possible to you to participate in what you would like to participate in? In what way does education/job impact on your quality of life and participation? How is your perception of Denmark as a country of residence when living with a disability?
Perception of body & health	Physical	Health and Functioning [62] Biological functioning, symptoms, functional status, general health perceptions [63] Physical wellbeing [46]	Which impact has your physical capacity on your quality of life and participation?
	Mental		Which impact has your mental capacity on your quality of life and participation?
	Treatment		What does medications and treatment mean to your quality of life and participation?
	Pain		How does pain affect your quality of life and participation?
Perception of personal factors	Future	Psychological/spiritual domain, health and functioning domain [62] Characteristics of the individual [63] Emotional wellbeing, material wellbeing, personal development, self-determination [46]	Please tell me about your dreams for the future (hope, fear, job situation, family)
	Coping		How do you handle your disabilities? What do you do when you face resistance?
	Desired perception		How would you prefer others to perceive you in relation to your disability?
	Dependence		How does it affect your self-worth when you are dependent/ independent of others?
	Economy		What does economic independence mean to you?
	Alcohol and drugs		Please share your thoughts about alcohol and drugs in relation to disability
	Sexuality		Can you tell me about sexuality and disability?

## Methods

### Step I: Establishment of conceptual understanding

The conceptual understanding was established through a thorough review of literature and on discussions within the author group. We took the approach that enhanced quality of life is a realistic and obtainable goal for all persons, including those with disability and chronic illness. We used the WHO definition as the basis for our understanding of quality of life: *“Individuals perception of their position in life in the context of the culture and value systems in which they live and in relation to their goals, expectations, standards and concerns”* [26]*.*

Our understanding of disability was inspired by the concepts of impairment and disability employed in the International Classification of Functioning, Disability and Health (ICF) [38]. The ICF is based on functioning rather than on diagnoses and provides a standard language for describing functioning and disability. Functioning encompasses body function, activities and participation whereas disability encompasses impairments, activity limitations and participation restrictions. The ICF shifted their focus from handicap to participation in 2001 [38] leading to more research on participation [39]. In this study, we defined participation as *involvement in a life situation* as presented by the ICF [38]. This definition address the way a person functions in society, for example in relation to education, social relations, and leisure activities such as hobbies or sports [9].

### Step II: Development of interview guides

Interview guides for focus group interviews were constructed based on the above described conceptual understanding [26, 38] and a framework on quality of life [40]. Broad open questions about the perception of the quality of life and participation concepts were listed in the beginning of the interview guide to allow the appearance of themes, which were not included in the interview guide. The concepts of environment, function and health and individual factors were included to explore how these were perceived to affect quality of life and participation. In an attempt to accommodate the different needs of the participants, we included exercises in the interview guides. For example, photographs to facilitate the discussions of similarities and differences in the perception of quality of life. The exercises have been described in detail elsewhere [41].

When interviewing young adolescents (10–12 years) and participants with intellectual disabilities an additional exercise was used, whereas questions about sexuality, impact of education/job and economic independence were omitted.

### Step III: Qualitative data collection, recruitment and study population

The data collection design was cross disability focus group interviews [42]. The intention with this design was that the variation in disabilities would stimulate the discussions by making explicit the similarities and differences that might be implicit to individuals with the same or similar disability. Inclusion criteria for focus group interview participants were that they had a self-reported disability (physical or mental chronic illness or impairment leading to a functional limitation), that they were between 10 and 40 years old at time of the interview, and that they were able to participate in an interview (but not necessarily able to answer a questionnaire). Proxy-respondents could be included to represent individuals with intellectual disability, who were unable to participate in a face-to-face interview.

In total, 55 people aged 10–40 years were included in the study. The group of participants was selected in order to represent as many disabilities and age groups as possible, aiming at an even distribution by gender. In total, we conducted 15 focus group interviews and 7 individual interviews, with individuals representing a broad range of disabilities.

The recruitment of potential participants included four steps.

- First, we approached participants by posting a short notice at Facebook inviting people with disabilities to participate in a focus group interview.
- Second, we contacted 34 patient/disability organizations by a personalized email to the director and we followed up via telephone calls. Following these initiatives, 32 organizations responded positively to our request. Letters including a short version of our invitation to interview participants were sent to all organizations except the two, which did not have the opportunity to participate. The invitation letter was appropriate for the organizations to publish on their webpage, Facebook page or in their membership magazine.
- Third, in order to recruit more young participants, we contacted a local school in Copenhagen with special classes, two leisure clubs for adolescents with special needs and 14 mainstream schools from the three main regions in Denmark; Jutland, Funen and Zealand. We offered to conduct the interviews at school premises.
- Finally, two participants were approached directly by email, after having appeared in the media discussing the issue of living with a disability.

**Fig. 2:**
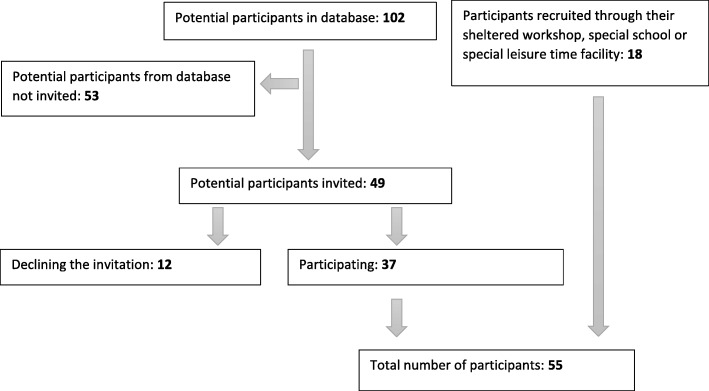
Inclusion of participants for focus group interviews and individual interviews

The recruitment of participants (Fig. 2) resulted in 102 potential participants with disabilities, who were included in a database with information on age, gender, type of disability and place of residence. Of the potential participants, 53 were not invited because the desired number of participants with that age and/or type of disability had already been attained and 12 declined the invitation for different reasons. Participants with intellectual disabilities were recruited through a contact person at their work place or leisure time facility.

Within each focus group, participants had the same gender and were similar in terms of age, but differed in type and severity of their disability. Three parents were included in a separate focus group interview as proxy-informants for people with intellectual disabilities, who the parents considered unable to speak for themselves.

Before each interview the participants were asked to answer a short questionnaire about age, gender, education, occupation and type of disability. All interviews were conducted by two of the authors (LNJ, SIM and a student assistant of whom two were present at each interview. The role of the one author was to facilitate the discussions initiated by the participants. Each interview was structured by the issues raised by the participants and therefore, the other author used the interview guide as a checklist, to ensure that all topics had been addressed. To allow our study design to be as inclusive as possible we additionally conducted seven individual interviews among people unable to attend focus group interviews. For instance, we interviewed a young woman with hearing loss assisted by a sign language interpreter, we interviewed a woman with multiple mental illnesses in a place she referred to as her ‘safe spot’, and we interviewed a young man without verbal language who communicated by way of his helper by spelling each word through blinking his eyes.

Information about the physical surroundings, the social dynamics in the group and the most dominant topics from each interview were documented in field notes during or immediately after each interview All interviews were audiotaped and partly transcribed. A summary of the perspectives, perceptions, agreements and disagreements was made for each question asked and to support the summaries, relevant quotes were fully transcribed.

### Step IV: Conceptualization

The conceptualization of turning the information provided by data from the qualitative interviews into domains to be included in the EQOL questionnaire was done stepwise.

#### Codes and themes

The first author and two student assistants independently coded each interview. This initial organizing of data was inspired by the first steps in thematic analysis [43]. A code had to be identified in more than one interview, before it was included as a code. Examples of codes were*, ‘understanding from others’, ‘wish to hide disability’ and ‘difficult to initiate a relationship’.* The codes from each interview were organized in themes each covering one or more codes. An example of a theme was *‘close social relations’*.

#### Empirical indicators and domains

**Fig. 3:**
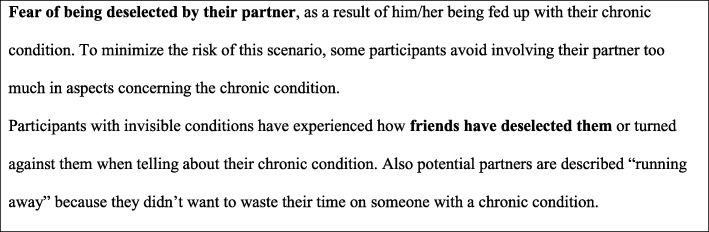
Example of a condensed content description of part of the wellbeing domain

Following conceptual discussions within the author group and with the external advisory board, the eight themes developed from the qualitative data were compiled in six domains to be included in the EQOL-questionnaire. A condensed description of each domain was generated to sum up the domain content. Figure 3 is an example of such a condensed content description of part of the wellbeing domain. To move closer to actual items, empirical indicators or *“not-quite-variables”* [31] were selected for each domain. These empirical indicators were chosen as one or a few words representing the essence of the domain (highlighted with bold text in Fig. 3). Domains as well as empirical indicators were included in a conceptual model (Fig. 5).

#### EQOL-model

We constructed a conceptual model of EQOL by relating each domain and their associated empirical indicators to the conceptual understanding (Step I). Each domain was included in the model as part of either the quality of life construct, the participation construct or as a combination of both (Fig. 5).

### Step V: Operationalization of empirical indicators into items

#### Items

Initial item formulations was made by one of the authors (LNJ) based on the condensed descriptions of the domains and based on the conceptual model. For each empirical indicator, the composed item(s) was constructed to reflect that specific aspect of the domain. As an example, the empirical indicator “*Fear of being left by partner*” was operationalized as follows: *Does your disability cause fear of being left by your partner/ potential partner?* Whenever possible, precise words or phrases used by participants in the interviews were applied. The item formulation and the original purpose of each empirical indicator were then compared and discussed in detail by the external advisory board and the author group. Subsequently, items were discussed and adjusted several times until consensus was reached within the author group, so that the item seemed to reproduce the content of the empirical indicator and that the items appeared relevant and understandable to the target group. The thoroughly defined domains (condensed descriptions) and their mutual relations (conceptual model) continuously guided the item development process. We used a few items from existing quality of life and participation questionnaires and the *Washington Group Short set of Questions on Disability* was included as a measure of disability [44]. All items were subsequently evaluated by the target group (step VI).

#### Response measures

When possible we used a frequency response measure: ‘Always/almost always’, ‘often’, ‘sometimes’, ‘seldom’, ‘never’ or a satisfaction measure: ‘Very satisfied’, ‘satisfied’, ‘neither satisfied nor dissatisfied’, ‘dissatisfied’, ‘very dissatisfied’. When a frequency response or a satisfaction response was not meaningful we used the response categories from a Likert scale: ‘Totally agree’, ‘agree’, ‘neither agree nor disagree’, ‘disagree’, ‘totally disagree’.

### Step VI: Evaluation of content validity

The content validity of EQOL was secured by means of the steps described previously: a conceptual understanding based on relevant literature, recruitment of participants representing a broad array of disabilities, conduction of a comprehensive data collection, and a transparent development of items.

Further the content validity was explored by cognitive interviews with participants in the target group and continuous evaluation and advocacy by experts in the field through the external advisory board.

#### Cognitive interviews to explore face validity

To explore how the target group understood and answered each item, we piloted the EQOL-questionnaire among nine participants from the target group. To encourage the respondents to comment, a comment box was added after each item. Seven items uncovering the experience of answering the questionnaire were added at the end of the questionnaire, e.g. how much time they used to answer the questionnaire and how well they liked the questionnaire.

After completing the questionnaire, we performed cognitive interviews with each participant about their assessment. Using retrospective thinking (uncovering what they were thinking while answering the question) [30], paraphrasing (how they would have phrased the question) [30], and probes (follow-up questions to uncover the rationale behind the given answers) [30], we investigated whether the participants assessed the items as covering quality of life and participation clearly and unambiguously [24].

### Ethics

Interview participants were informed orally and in writing about the aim and scope of the study, that their participation was voluntary, and that they could withdraw at any time without consequences. For young participants (under 18 years), informed consent was also obtained from their parent or legal guardian. Participants were further informed that data was treated as confidential information. Since the participants are in a vulnerable situation, we made great efforts to ensure that they felt comfortable during the interview process, that they felt inspired and confident to give voice to their situation and concerns, and that the interview situation did not violate their personal boundaries. The study complies with the ethical guidelines of the Declaration of Helsinki [45] and was approved by the Danish Data Protection Agency. Registration number: 17/3455.

## Results

### Results from step I and II: Establishment of conceptual understanding and development of interview guide

The conceptual understanding provided a basis for the development of the interview guide including five broad concepts and 18 sub-concepts as illustrated in Table 1. These concepts provided the foundation for developing the EQOL-questionnaire based on existing literature.

### Results from step III: Qualitative data collection

More than 40 different diagnoses were represented among the participants. Some participants had identical diagnoses, and many reported multiple diagnoses. Some participants reported all of their diagnoses, others only reported what they considered to be the most important reason for their disability and most participants with intellectual disabilities did not report any diagnosis or disability.

Of the participants 55% were female, 11% were between 10 and 12 years old, 15% were between 13 and 15 years old, 20% were between 16 and 19 years old, 36% were between 20 and 29 years old, and 18% were between 30 and 40 years old. A table of the demographic characteristics and the disabilities reported by the participants is presented elsewhere [41]. Examples of dominant topics across interviews, as documented in field notes, were: *The social system and its impact on quality of life* and *how the function of social relations was different when living with a disability.*

### Results from step IV: Conceptualization

A data matrix was constructed as the first step in the conceptualization of the qualitative data. Related codes from the interviews were placed in the same row creating a starting point for the generation of themes. As an example of this matrix, Table 2 shows the codes identified in the environment theme. The codes show how *environment* was articulated and discussed in the first four focus group interviews. As an example, accessibility was only directly addressed in interview number 4 (Table 2, row 1).

**Table 2:** The environment theme as articulated in the first four interviews. Each row represents a sub-theme

Interview 1	Interview 2	Interview 3	Interview 4
			Accessibility
How much I choose to share about my illness The importance of age Prejudice Avoid telling it-if possible	Judgemental	The possibility of hiding your illness Misjudged considerations by teachers -feel exposed	Being pigeonholed Lack of understanding
Mistrust Disrespecting that they interfere Exhausting to apply for aids Being reminded about the illness A wish for a solution-orientated approach	Interchanging social workers They believe you to be ill just for fun Inflexible You expose yourself, but there is a lack of understanding	Irrelevant offers A wall of bureaucracy	Mistrust Lack of time/money- cutbacks Unprepared social workers Being nothing but a number Lacking information about rights
Often only medical solutions are offered. The rest you need to look for yourself. Lack of information regarding prevention Difficult to obtain continuous controls after turning 18 years. Wish for alternatives to medication	Medications as the only suggestion Medication is good -if it helps and bad if it brings adverse reactions	Lack of confidence in your general practitioner Announcing that you have to learn how to live with it. The professional patient (responsibility for your own treatment)	Lack of stable and continuous contact to doctors. Medication is good and bad Side effects as limitations e.g. in relation to alcohol.
Avoiding the illness by being with others having the illness Feeling understood and accepted among others who are ill	Relief to be with others who are ill Misunderstood sympathy Periodic needs Too much illness when being with others who are ill	Relief not having to explain	

**Fig. 4:**
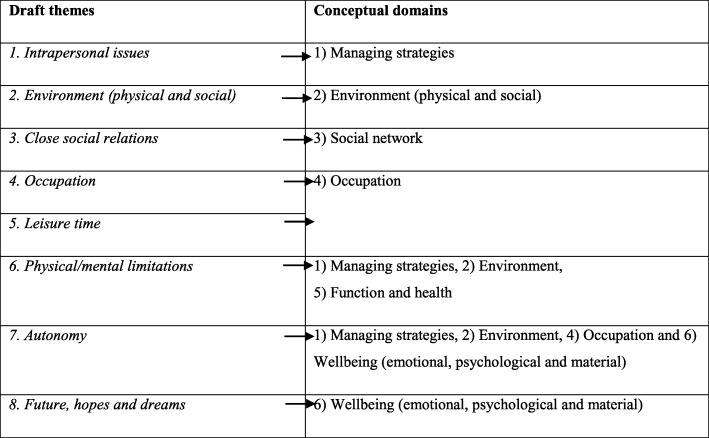
Overview over the conceptual domains derived from qualitative themes

The structure of codes in the data matrix guided the generation of themes by looking at similarities and differences between the codes. For each of the interviews, the codes indicate in which way the theme was addressed or discussed. The final matrix structure was subsequently examined to get an idea of the perceived importance of each code and theme. We found, that some codes were crucial to a small group of participants. Thus, a frequent appearance of a code did not necessarily mean that we considered it to be more important than less frequently appearing codes [43]. A draft list of eight themes from the matrix was generated (Fig. 4, first column). Following discussions with the advisory board, six domains (Fig. 4, second column) were constructed from the eight themes: The theme of intrapersonal characteristics became a domain of *managing strategies*. The theme of social relations was split into two domains: *social network* and formal relations, which were included in the *environment* domain representing the social environment. An *occupation* domain was expanded to include leisure time. Codes within the theme of physical and mental limitations were split between domains of *managing strategies*, *environment*, a new domain called *function and health* and background information. Future, hopes and dreams were included in a new domain of *wellbeing* (Fig. 4).

#### Conceptual model

**Fig. 5:**
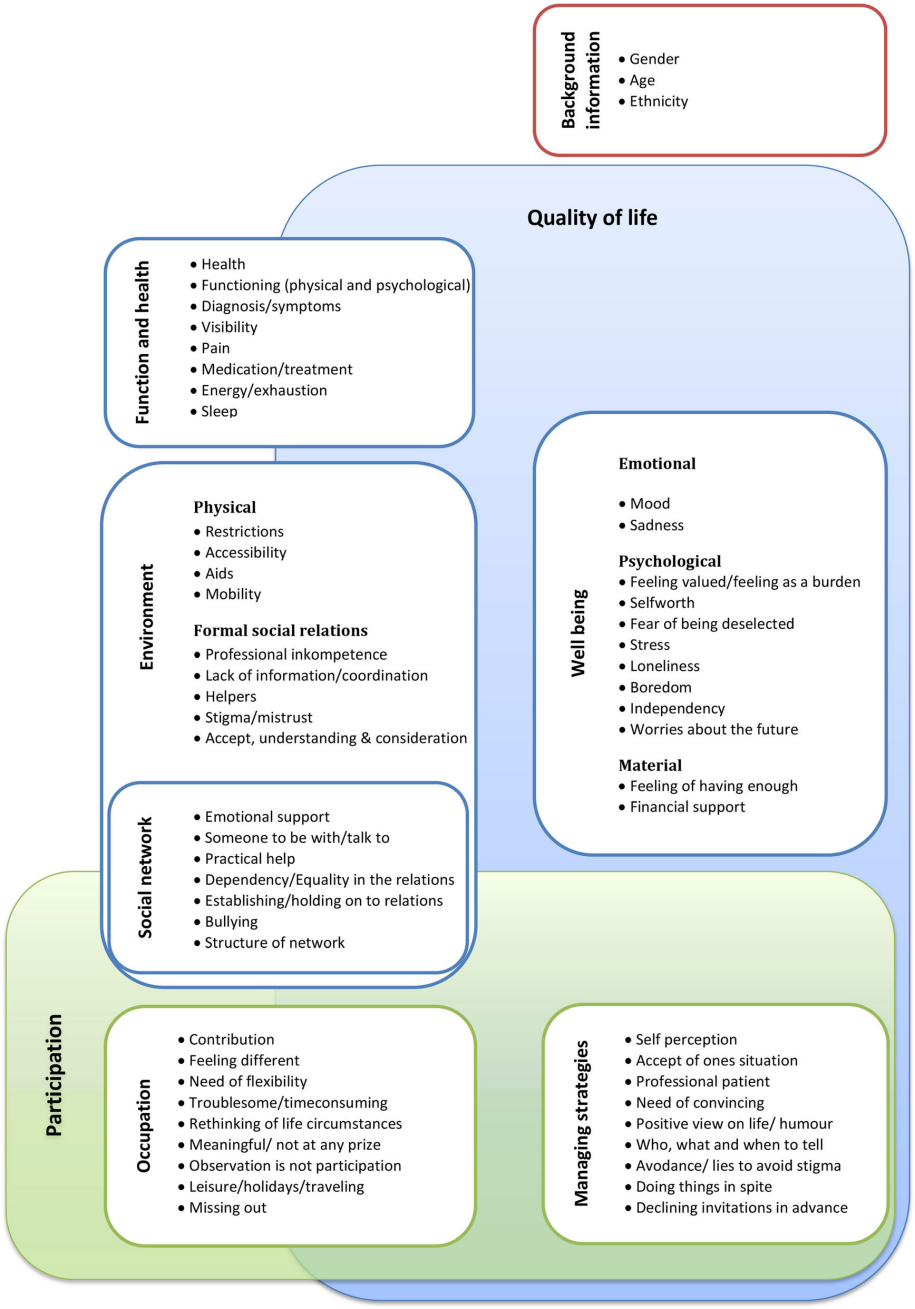
Conceptual model with incorporated domains and empirical indicators

The six domains: *function and health, environment (physical and social), social network, wellbeing, occupation, and managing strategies* were incorporated in a conceptual model (Fig. 5) covering the domains and empirical indicators which participants across age and diagnoses perceived as important to their quality of life and participation. The vertical dimension illustrates quality of life and the horizontal dimension illustrates participation. The intersection between the two dimensions indicates a conceptual overlap between the content of the domains. Domains that are placed partly outside the dimensions illustrate that the domain also includes more objective aspects of the concepts (for example whether or not you take medications). The box to the top right shows the included background information.

### Results from step V: Operationalization

The aim of the operationalization was to transform the complex content of the domains into items which could be answered by questionnaire respondents. The items developed for each domain was in accordance with the conceptual model (Fig. 5). Each empirical indicator provided the basis for development of items to measure specific parts of the domain. If necessary, more items were constructed to cover each empirical indicator. This was for example true when items needed to be related to different family members (e.g. parents, siblings, partner) or to different occupation (e.g. student, employed, unemployed).

Based on the domain of managing strategies, 20 items were developed to measure quality of life and participation related to personal disability management. The environment domain (physical and social) resulted in the development of 21 items including objective items and perceptions of quality of life in relation to the environment. The social network domain was located in the intersection of the quality of life and participation dimensions (Fig. 5). Therefore, the 35 items developed for this domain were objective, participation-related (what you do with your social network), and quality of life-related (how you feel about your social network). In the occupation domain, 27 items were developed to measure things you do (participation) and the feelings connected to the things you do (quality of life). In total, 40 items were developed to determine participants’ objective health and functional status, as well as their quality of life in relation to their health and functional status. The wellbeing domain was purely subjective and measured by 41 questions related to how one feels.

A total of 191 items covering six domains, were included in the EQOL questionnaire.

We separated the items in a generic section with 116 items and a chronic-generic section with 75 items. The items in the generic section could be answered by everyone able to self-report and did not include items concerning disability. Items in the chronic-generic section referred to aspects of disability identified in the qualitative interviews as important to quality of life for some or all individuals with disabilities.

### Results from step VI: Evaluation of content validity

#### Face validity

Nine participants from the target group were asked to answer the draft questionnaire and share their assessments of the EQOL-questionnaire with us. Overall the participants had a positive attitude towards the questionnaire and found the constructs of quality of life and participation to be clearly and unambiguously covered. One participant expressed that this was the first time she felt accommodated in a questionnaire. Another respondent was satisfied that it was possible for him to provide answers to questions which he found to be important to his quality of life but rarely asked. As examples were questions about feeling different from others and worries that his disability might affect his chances to get a job. On the negative side, most participants felt that there were too many questions and that some questions were irrelevant to them, for example questions about accessibility for participants who did not feel that their access was limited.

Some respondents expressed confusion about the term ‘family’ because there were large deviations in their preferred answers when relating to close and extended family respectively. As an example, a respondent explained how she felt valued and understood by her partner and her children, but felt misunderstood by her parents. A child and two participants with intellectual disabilities answered a reduced number of questions excluding the most complex questions. They found some of the questions challenging and others too difficult to answer while the less abstract questions worked well. Participants did not agree on which type of response categories they preferred, but generally, the response categories were perceived to be satisfactory.

During focus group interviews and cognitive interviews, we discussed which word(s) to use when referring to disability. We became aware that participants preferred to use different words to describe their disabilities and illnesses. As an example, participants with minor disabilities such as allergies found that *functional limitations* didn’t apply well to their situation. Participants with physical conditions perceived *functional limitations* to be more applicable than participants with mental conditions.

In accordance with the feedback from the respondents we adjusted the layout (e.g. added more space between the response options) and added a few items on new subjects. For example, we added a question about feeling left out because you were only partly able to participate (e.g. not being able to eat the same as others because of allergy or having to sit somewhere else due to use of a wheelchair). Most participants in the face validity interviews found that some of the questions were irrelevant to them. This issue is difficult to completely avoid in a cross-disability questionnaire since items that are highly relevant to some are irrelevant to others. To accommodate this feedback from the target group, we introduced more tailored questions to reduce the number of irrelevant questions.

#### Expert evaluation

The draft questionnaire and the conceptual model were sent to the advisory board to get their assessment of the coverage of constructs. Seven board members provided their feedback and pointed out some potential problems with the measure of disability; the advisory board commented that people with the same disability could provide different answers according to their access to aids and help since the questions were formulated: “Do you have any difficulty..”. Therefore, this assessment of functioning would be a measure dependent on the environment rather than an objective measure of functioning. Nevertheless, we could not identify a better measure of functioning and therefore decided to keep the items.

Following the feedback from the advisory board, adjustments were made. Examples of these adjustments are: More text was added to the questionnaire to indicate the transition from one domain to another. How often you were *together with* others was changed to how often are you *in contact* with others to allow for contact through the Internet or telephone. Initially, we had used the word *diagnosis* to refer to disabilities, functional limitations and illnesses. This was changed to *functional limitations* by request from the advisory board.

## Discussion

This study summarizes the initial steps in the development of the EQOL-questionnaire measuring quality of life and participation across persons with diverse mental and physical disabilities. We identified six domains of quality of life and participation common to persons with diverse disabilities. The six domains; *function and health, environment (physical and social), social network, wellbeing, occupation and managing strategies* were incorporated in a conceptual model. A questionnaire with 191 items was developed to cover the domain content.

### Step I and II

Using the WHO-definition on quality of life, we addressed quality of life subjectively. This view on quality of life is supported in the literature [9, 26]. However, some inconsistency exists as objective aspects of quality of life are included in several definitions, frameworks [40, 46] and quality of life models [47]. It has been argued that what is considered objective measures are not really objective, but rather normative decisions of what contributes to a high quality of life [48]. For example, does having a job, a high income, or owning your own house always lead to improved quality of life? [49]. Further, a taxonomy of quality of life argues that the objective quality of life measures often make use of absolute standards, which may make comparison across cultures (or even across different groups within the same culture) problematic [50].

The themes and questions included in the interview guide were inspired by the ICF and a conceptual framework on quality of life [40]. The inclusion of established theory was a strength, since the questionnaire was positioned in relation to the existing ‘nomological net’ of quality of life concepts and causal paths [51]. Hagety et al. (2001), reviewed 22 of the most frequently used quality of life indexes and concluded that most of the indexes failed to define any theoretical basis [51].

### Step III

The target population in this thesis was participants with a wide range of disabilities including individuals with mental and intellectual disabilities and communication difficulties, who tend to be excluded from other research [13, 52]. The recruitment was challenging, which is in accordance with the literature showing that identification and recruitment within a minority population is difficult [53]. We made several efforts to recruit more children and men to the focus group interviews. But despite of this, we ended up with fewer children than we planned.

The focus groups were used as a method of gaining knowledge on shared aspects of quality of life and participation to be able to construct relevant domains for the EQOL-questionnaire. This is a commonly used method to guide the content of a questionnaire [54] and have been found to be especially helpful in settings where perspectives of the target group and the researcher differ or where little is known about the target group [54]. Due to the wide-ranging group of participants, we were constantly aware that the interviews might turn out differently and we were prepared to adjust our plans accordingly during the interviews.

The cross disability approach applied in the development of EQOL is in accordance with previous research. Already in 1989, Stein and colleagues introduced a non-categorical framework in a study of 209 children from 0 to 11 years old. They found that diagnosis were only related to the traditional medical variables and suggest that when moving from the biomedical view toward a more psychosocial view, diagnoses do not provide sufficient information on the status of the child [55]. This support the need for a quality of life measure that is applicable across diagnoses.

A similar broad approach has been used in the development of the DISABKIDS-questionnaire [56] which is developed specifically to meet the needs of children living with different chronic diseases or disabilities. In this study, we implemented such a non-categorical approach in two ways: 1) By including participants with diverse types and severity of disability: 2) By asking participants with multiple disabilities to reflect on their overall impact of their disabilities on their quality of life. Further, we included individuals with intellectual disabilities and individuals without verbal language in our study, individuals who tend to be excluded from interview studies [52].

The proxy responses comprised in this study were used to ensure the inclusion of perspectives representing the entire range of disabilities. We are aware that the proxy responses cannot be ranked alongside the subjective responses, and proxy responses will not be included in the subsequent field tests nor the psychometric analyses.

### Step IV

The themes identified in the qualitative interviews informed the six domains included in the EQOL-questionnaire. At a glance, the EQOL-domains are in accordance with previous research, which have reached consensus on the multidimensional nature of quality of life, including at least a physical, a psychological, and a social dimension [40, 46, 47, 51]. Generally, the EQOL-domains seem to match the research on important quality of life domains as identified in *The conceptual model of quality of life* by Felce and Perry (1995) [47] and *the conceptual framework* by Schalock (2004) [40, 46]. However, differences appear when domains are elaborated. The differences are mainly due to aspects being placed in different domains or when different names have been chosen to describe the domains or aspects. However, a few differences require attention. Managing strategies were covered in much more detail in the EQOL-model than in the two reviews. For example, the additional burden of constantly having to convince others about the sincerity of your limitations or capabilities was not found as a common theme in any of the two domain collections. We found no obvious explanation for these differences. Legal rights e.g. due process was not addressed in the EQOL-model. An explanation for this might be cultural differences. It has been argued that populations in different cultural settings perceive the importance of domains differently, and that a ‘leisure’ domain may only be perceived important in developed countries [51]. The missing attention given to the legal rights in the EQOL-model may be explained by the fact that the identified empirical indicators were perceived more important than the legal rights in Denmark. This may also explain the missing aspect of physical safety and religious faith in the EQOL-model. This potential difference in relation to culture is also clear in the WHO definition of quality of life *“Individuals’ perception of their position in life in the context of the culture and value systems in which they live and in relation to their goals, expectations, standards and concerns”* [26] ^*p 1570*^*.*.

### Step IV

When trying to fit reality into ‘boxes’ (domains), there is always a risk that things would have been better suited for another domain, or maybe in more than one domain. It has been suggested that imperfections in definitions of quality of life may be a consequence of trying to include almost all aspects of life in one definition [57]. This suggestion is also likely to apply to the challenges in conceptualizing and operationalizing almost all aspects of human life. We strove to make the process as clear as possible by providing information on why the empirical indicators were included in the specific domains. Despite the conceptual discussions, there seems to be agreement on the importance of avoiding confusion of the concepts by being clear and precise about the definitions used [50]. To accommodate the discrepancies in the existing definitions of quality of life, we strived for transparency in the development of the EQOL-questionnaire, allowing others to comment on the conceptualization and operationalization which provided the basis for the questionnaire.

In accordance with Hox [31] we found that the conceptualization process yielded clarity to the content of each domain. Furthermore, the condensed description facilitated the possibility of including words, expressions and phrases used in the focus groups in the item development. This may improve the validity of the results from the EQOL-questionnaire [58]. The development of empirical indicators helped indicating the number of items needed to cover the domains.

### Step V

Although a large part of the items is generic, the intention was not to construct a comprehensive measure of quality of life for all. Rather, the intention was to allow for comparison between people with and without disabilities on the aspects of quality of life that were found to be relevant to people with disabilities. It is a known challenge to construct a questionnaire containing all aspects of perceived importance, which at the same time is short and quick to use in practice [59]. Although we strived to reduce the number of items by tailoring the questionnaire, the EQOL-questionnaire was still extensive.

### Strengths and limitations

The thorough identification of themes meaningful to persons with diverse physical and mental disabilities enables the EQOL-questionnaire to measure quality of life across disabilities. Our cross-disability approach makes it possible to measure the potential impact of having multiple disabilities on quality of life and participation. To our knowledge, no other studies have empirically identified common themes of quality of life and participation in a large group of interview participants representing such wide range of disabilities. However, the cross-disability approach may also be a potential weakness when participants would have preferred to provide different answers depending on which of their multiple disabilities was addressed [60]. Further, the heterogeneity in types of disability may be a limitation affecting the specificity of the questionnaire.

The use of predefined concepts in the interview guide may also be a limitation of our study, as these may have blinded us to aspects of data not included in the interview guide. However, the guide was not followed rigorously and the interview structure allowed for discussions initiated by the participants.

The small number of participants involved in face validity interviews and the large number of items are other potential limitations of the EQOL-questionnaire. However, a potential use of the questionnaire is in sheltered accommodations where participants may be more willing to answer an extensive questionnaire knowing that their answers will be used to improve their living conditions.In its current form, EQOL is applicable only in a Danish setting, as the development is based on a Danish population and reflects service settings and provision in Denmark. However, it is our perception that the obtained qualitative insight and conceptual work can inspire others who wish to address quality of life in a cross-disability perspective.

### Implications for practice and future research

The EQOL-questionnaire provides a new instrument for measuring quality of life and participation across disabilities.

To improve the content validity of a child version of the questionnaire, more tests on children are needed. Further we recommend a stronger focus on children by including more children in future studies. Two individuals with intellectual disabilities participated in the pilot test, but needed so much guidance and explanation on the items, that we considered them unable to answer the items without extensive help. This calls for the development of a version of the questionnaire with fewer response options and/or symbols supporting the response options. We hope to develop an adapted version of the questionnaire, making it possible for individuals with intellectual disabilities to answer the questions independently, and a proxy-version for carers of those with intellectual disabilities unable to answer the questionnaire themselves. We recommend others to standardize the feedback from the content validity interviews. For instance, by using the COSMIN checklist [61].

A field-test and subsequent psychometric evaluation is recommended as the next step in the validation of the EQOL-questionnaire. Analysis of differential items functioning (DIF) should be used to evaluate whether items may measure something different for different subgroups (for example participants with visible disabilities and participants with invisible disabilities). To explore the convergent validity of EQOL, we suggest to use WHOQOL-dis for quality of life across different types of disabilities [26]. Further, confirmatory factor analysis could be applied to investigate the factor structure and the model fit.

EQOL was developed for use in a Danish population but may be applicable in other North European settings. However, translation and back-translation should be performed and face validity and field test should be conducted before applying the questionnaire in other settings.

Although the age rage for this study was limited to 10–40 years old we find, that there is a potential to expand the range to 65 years, which is the retirement age in Denmark. To confirm this, a few additional focus groups should be conducted to ensure that the perception and experiences of the entire age group are included or can be added in the questionnaire.

It is an important assignment to develop algorithms for calculation of domain specific EQOL indices. Furthermore, we suggest to develop a short version of the EQOL-questionnaire. It is our hope that EQOL can prompt interventions targeted at specific factors that can improve quality of life and participation in the target group. We suggest that the EQOL-questionnaire can be used in Danish settings where individuals with various types of disabilities are represented. This could for example be sheltered accommodations, sheltered workshops or in hospitals.

## Conclusion

We developed the EQOL measure based on qualitative interviews with participants with diverse disabilities. Six domains were constructed from the themes derived from the qualitative interviews (*function and health, environment (physical and social), social network, wellbeing, occupation, and managing strategies*). In total, 191 items were included in the EQOL-questionnaire and separated in a generic section (116 items) and a chronic-generic section (75 items).

Although evaluation of the psychometric properties is needed, we have shown, that it is possible to assess quality of life and participation in people with various diagnoses and functional limitations with a chronic generic questionnaire Although capturing less detail than a condition specific questionnaire EQOL includes aspects perceived important for people with disabilities that are not included in general surveys. This is highly relevant when for example evaluating environmental adaptations or services and when comparing populations with various disabilities.
